# A 14-Year-Old Boy with Torsion of the Epididymal Cyst

**DOI:** 10.1155/2015/731987

**Published:** 2015-12-21

**Authors:** Mojtaba Ameli, Samaneh Boroumand-Noughabi, Leila Gholami-Mahtaj

**Affiliations:** ^1^Department of Urology, Faculty of Medicine, Gonabad University of Medical Sciences, Gonabad, Iran; ^2^Mashhad University of Medical Sciences, Mashhad, Iran; ^3^Gonabad University of Medical Sciences, Gonabad, Iran

## Abstract

Epididymal cyst is a benign mass in the scrotum that is relatively common in adults but it is rare in children. In routine experience the treatment of such cysts is conservative. Torsion of these cysts is extremely rare and the diagnosis is made by exploration of the scrotum. Our patient was a 14-year-old boy who has been referred to hospital with scrotal pain followed by a minor trauma 3 days ago. Exploration of the scrotum to rule out testicular rupture was performed and a large black cyst connected to the head of the epididymis with 720-degree rotation was found. The cyst was resected and pathologic examination revealed an acquired epididymal cyst (spermatocele). The patient has normal physical exam after 3 months' follow-up.

## 1. Introduction

Epididymal cyst is a benign mass that is relatively common in adults but it is rare in children and its prevalence in children is 5–20% [1]. When it has been diagnosed certainly using ultrasound, the treatment of these cysts is conservative under elective condition. But in rare circumstances, as a result of trauma or torsion of these cysts, exploration of the scrotum is needed to rule out other pathologies such as testicular torsion. Torsion of an epididymal cyst is extremely rare especially in young boys and to the best of our knowledge only 5 cases have been reported in literature [2].

## 2. Case Presentation

**Figure 1:**
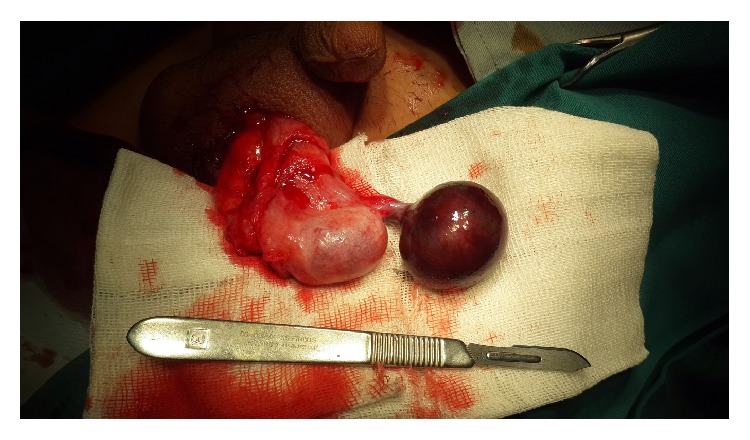
Intact testis at the left side and twisted cyst at the right side are seen.

**Figure 2:**
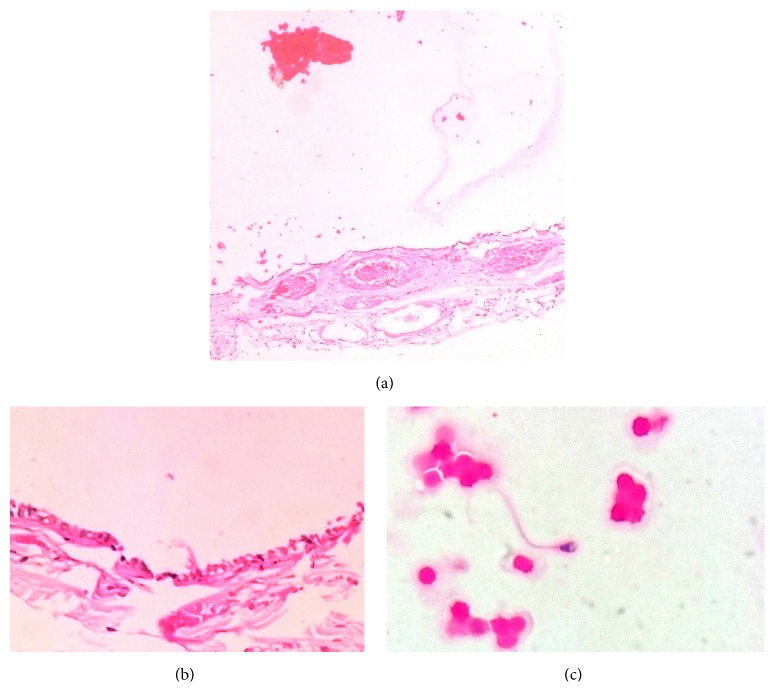
(a) and (b) Low and high power views of the cyst wall. The cyst wall is congested and hemorrhagic and is lined by ciliated low columnar cells (H&E, ×100 and ×400, resp.). (c) Cytological appearance of the cyst fluid. A spermatozoon surrounded by red blood cells is demonstrated (H&E, ×400).

The patient was a 14-year-old boy who has been referred to hospital with erythema, swelling, and pain in left hemiscrotum. The patient had a history of minor trauma to scrotum 3 days ago. He had no fever or urinary symptoms. Physical examination demonstrated tenderness and swelling of the left hemiscrotum. The margins of left testis were vague but the right testis was normal. Urine analysis was unremarkable. Color Doppler Ultrasonography showed a heterogenic area in the upper pole of the left testis. Based on history and physical examination, patient was transferred to operating room with the diagnosis of testicular trauma. After exploration of tunica vaginalis, a large black cystic mass (4 × 4 × 3 cm) connected to the head of epididymis which was twisted for 720 degrees was seen. This cyst has a pedicle on the head of epididymis (Figure 1). As obviously shown in the picture, the testis was normal. On opening, the cyst contained yellowish brown semiclear fluid which has been demonstrated to contain spermatozoa in microscopic examination (Figure 2). The cyst was composed of a fibrous wall with congested blood vessels and areas of hemorrhage and necrosis. It was lined partially by ciliated cuboid to columnar cells (Figure 2). The patient was discharged one day after surgery and physical examination was normal during 3 months' follow-up.

## 3. Discussion

Acute scrotum is a urologic emergency. The etiologies are including testicular torsion, epididymorchitis, torsion of testis appendix, trauma, and hernia. In cases in which ultrasonography could not rule out the diagnosis of testicular torsion or rupture, exploration of scrotum is indicated. Treatment of epididymal cysts is conservative unless in case of huge size of the cyst or patient preference [1].

Acute scrotum in these cysts (i.e., torsion of epididymal cyst or spermatocele) is extremely rare [1–4]. As the sudden onset of symptoms and the presence of tenderness in physical examination that is similar to testicular torsion, exploration of the scrotum is indicated. However, in our patient, due to the history of minor trauma and heterogeneity of the upper pole of testis in ultrasonography, exploration was done to rule out testis trauma. The size of cyst in our patient was larger than similar reports [1, 2] and the cyst was larger than patient's testis. In addition, our patient had no medical complaint for his cyst before surgery. However, in previously reported cases, only one had preoperative diagnosis of epididymal cyst [1, 2].

As cases of testicular torsion in association with epididymal cyst have been reported [5], it is important that patients with epididymal cysts, who are treated conservatively, be educated for warning signs that are including testicular sudden pain, erythema, and swelling of scrotum. So torsion of epididymal cyst is not the only diagnosis in these patients. In this case, the cyst was big and obvious. But in cases where the cyst is less conspicuous, the surgeon should make attempt to look for this entity if the testis is clearly not the cause of an acute scrotum.

## 4. Conclusion

Epididymal cysts may twist as the testis torsion and may cause symptoms exactly similar to testicular torsion. Torsion of epididymal cysts should be considered in differential diagnosis of acute scrotum.
